# Allosteric Inhibition of Phosphoenolpyruvate Carboxylases is Determined by a Single Amino Acid Residue in Cyanobacteria

**DOI:** 10.1038/srep41080

**Published:** 2017-01-24

**Authors:** Masahiro Takeya, Masami Yokota Hirai, Takashi Osanai

**Affiliations:** 1 School of Agriculture, Meiji University, 1-1-1, Higashimita, Tama-ku, Kawasaki, Kanagawa 214-8571, Japan; 2 RIKEN Center for Sustainable Resource Science, 1-7-22 Suehiro-cho, Tsurumi-ku, Yokohama, Kanagawa 230-0045, Japan

## Abstract

Phosphoenolpyruvate carboxylase (PEPC) is an important enzyme for CO_2_ fixation and primary metabolism in photosynthetic organisms including cyanobacteria. The kinetics and allosteric regulation of PEPCs have been studied in many organisms, but the biochemical properties of PEPC in the unicellular, non-nitrogen-fixing cyanobacterium *Synechocystis* sp. PCC 6803 have not been clarified. In this study, biochemical analysis revealed that the optimum pH and temperature of *Synechocystis* 6803 PEPC proteins were 7.3 and 30 °C, respectively. *Synechocystis* 6803 PEPC was found to be tolerant to allosteric inhibition by several metabolic effectors such as malate, aspartate, and fumarate compared with other cyanobacterial PEPCs. Comparative sequence and biochemical analysis showed that substitution of the glutamate residue at position 954 with lysine altered the enzyme so that it was inhibited by malate, aspartate, and fumarate. PEPC of the nitrogen-fixing cyanobacterium *Anabaena* sp. PCC 7120 was purified, and its activity was inhibited in the presence of malate. Substitution of the lysine at position 946 (equivalent to position 954 in *Synechocystis* 6803) with glutamate made *Anabaena* 7120 PEPC tolerant to malate. These results demonstrate that the allosteric regulation of PEPC in cyanobacteria is determined by a single amino acid residue, a characteristic that is conserved in different orders.

Cyanobacteria are a group of bacteria that perform oxygenic photosynthesis and fix carbon dioxide. Ribulose-1,5-bisphosphate carboxylase/oxygenase (RubisCO) is the most famous CO_2_ fixing enzyme, which operates in the Calvin-Benson cycle^1^,^2^. Besides RubisCO, metabolic flux analysis revealed that phosphoenolpyruvate carboxylase (PEPC) [EC 4.1.1.31] accounts for 25% of CO_2_ fixation in the unicellular cyanobacterium *Synechocystis* sp. PCC 6803 (hereafter *Synechocystis* 6803)^3^. PEPC is a crucial branch point enzyme determining the type of carbon fixation in photosynthetic organisms^4^. PEPC catalyses an irreversible carboxylation of phosphoenolpyruvate (PEP) with bicarbonate (HCO_3_^−^) to generate oxaloacetate and inorganic phosphate in the presence of Mg^2+^ 4. PEPC is conserved among plants, algae, cyanobacteria, archaea, and heterotrophic bacteria, but not among animals, fungi, and yeasts^5^. Cyanobacterial PEPC also plays an anaplerotic role in energy storage and biosynthesis of various metabolites by replenishing oxaloacetate to the citric acid cycle^5^.

The kinetics of PEPCs are diverse among organisms. Higher plants can be classified as C3-type, C4-type, and crassulacean acid metabolism (CAM) plants. PEPC is responsible for the primary carbon fixation in C4-type and CAM plants^6^,^7^. The affinity of PEPCs in C4-plants to bicarbonate is 10 times higher than that of PEPCs in C3-plants^8^,^9^. Most PEPCs are allosterically regulated by various metabolic effectors. Maize PEPCs are inhibited by malate or aspartate, and activated by glucose-6-phosphate^10^. *Escherichia coli* PEPC is inhibited by malate or aspartate, and activated by acetyl-CoA^11^. Cyanobacterial PEPCs are evolutionally diverse. One group has suggested that PEPCs of the orders *Oscillatoriales* and *Nostocales* (including the nitrogen-fixing cyanobacterium *Anabaena* sp. PCC 7120, hereafter *Anabaena* 7120) resemble C4-type PEPC because of the serine residue conserved among C4 plants at position 774^12^. However, subsequent sequence analysis has revealed that most PEPCs contain the conserved serine residue; nevertheless the kinetic properties of cyanobacteria PEPCs are diverse^12^. Therefore, there may be a different type of regulation in cyanobacterial PEPCs. Cyanobacterial PEPCs in the order *Nostocales, Coccochloris peniocystis*, and *Thermosynechococcus vulcanus* are inhibited by either malate or aspartate^12^,^13^,^14^,^15^. Several effectors regulate cyanobacterial PEPCs, but their effects are dependent on the taxonomic order of the PEPCs^12^. The biochemical properties, including *V*_max_ and *K*_m_ values, of several cyanobacterial PEPCs have been determined^12^,^14^,^15^, although those of the PEPCs in *Synechocystis* 6803 have not. A comparison of cyanobacterial PEPCs including both phylogenetic and biochemical analyses has also been lacking until now.

Here, using the model cyanobacterium *Synechocystis* 6803, we performed biochemical analysis using purified PEPC proteins. Our analysis demonstrated that a single amino acid substitution between glutamate and lysine at position 954 was important for allosteric regulation.

## Results

### Measurement of the kinetic parameters of and inhibitor effects on *Synechocystis* 6803 PEPC

**Figure 1:**
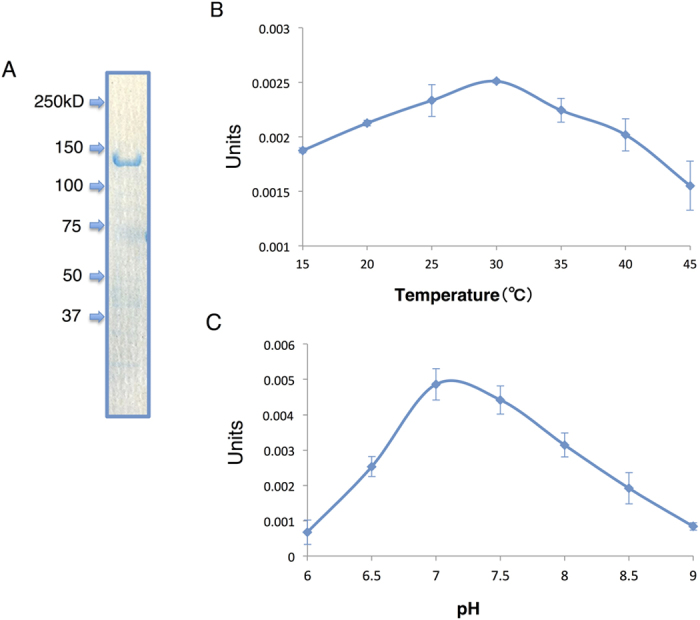
Biochemical analysis of *Synechocystis* 6803 phosphoenolpyruvate carboxylase (*Sy*PEPC). (**A**) Purification of GST-tagged PEPC. Proteins were electrophoresed on an 8% SDS-PAGE gel, and stained with Instant Blue reagent. Arrowheads indicate the molecular weight. (**B**) Effect of temperature on *Sy*PEPC activity. Data represent means of the values from three independent experiments. (**C**) Effect of pH on *Sy*PEPC activity. Data represent relative values of means from three independent experiments. Four pmol (0.6 μg) of *Sy*PEPC was used for the enzyme assay. One unit of PEPC activity was defined as the consumption of 1 μmol NADPH per minute.

**Figure 2:**
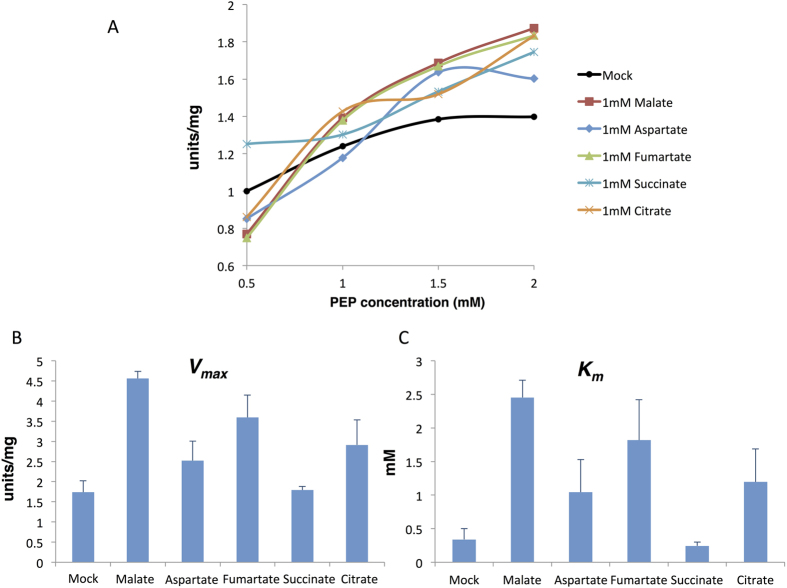
The *V*_max_ and *K*_m_ values for phosphoenolpyruvate (PEP) in the presence of various compounds. (**A**) Saturation curves of the activity of purified *Sy*PEPC. The graph shows the means of three independent experiments. The *V*_max_ and *K*_m_ values for PEP of GST-tagged *Sy*PEPC proteins are shown in (**B**) and (**C**), respectively. (**B**) Mean ± SD *V*_max_ (units/pmol protein) values in the presence of various compounds, obtained from three independent experiments. (**C**) Mean ± SD *K*_*m*_ values for PEP, obtained from three independent experiments. Mock indicates the enzymatic activity in the absence of additional compounds. One unit of PEPC activity was defined as the consumption of 1 μmol NADPH per minute.

*Synechocystis* 6803 is one of the most studied cyanobacteria; nevertheless, the kinetic parameters of *Synechocystis* 6803 PEPC (*Sy*PEPC) have not been determined until now. Glutathione *S*-transferase (GST)-tagged *Sy*PEPC proteins were expressed in *E. coli* and purified by affinity chromatography (Fig. 1A). The enzymatic activity of *Sy*PEPC was highest at pH 7.3 and 30 °C (Fig. 1B and C). Biochemical analysis revealed the *V*_max_ value of *Sy*PEPC was 1.74 units/mg, and the *K*_m_ values of *Sy*PEPC for PEP and HCO_3_^−^ were 0.34 and 0.80 mM, respectively (Fig. 2A).

We next examined the effects of various metabolic effectors on *Sy*PEPC activity. The enzyme assay was performed at the optimal pH 7.3 and temperature 30 °C using a half-saturating concentration of PEP. Aspartate decreased the *Sy*PEPC activity to 85.2% (Table 1). The tricarboxylic acid cycle (TCA) metabolites malate, fumarate, and citrate reduced the *Sy*PEPC activity to 75–86% (Table 1). Both malate and fumarate increased the *V*_max_ and *K*_m_ values for PEP (Fig. 2B and C).

To strengthen the integrity of our results, we performed biochemical assays using commercially available PEPCs and cell extracts from other organisms. The purified PEPCs of *Acetobacter* and *Zea mays* were inhibited by both aspartate and malate (Fig. S1A). The activity of PEPCs in *Nostoc* sp. NIES-3756 and *E. coli* DH5α extracts were decreased by both aspartate and malate (Fig. S1B). These results were consistent with previous results^12^,^16^,^17^, confirming our data were reliable (Fig. S1C).

**Figure 3:**
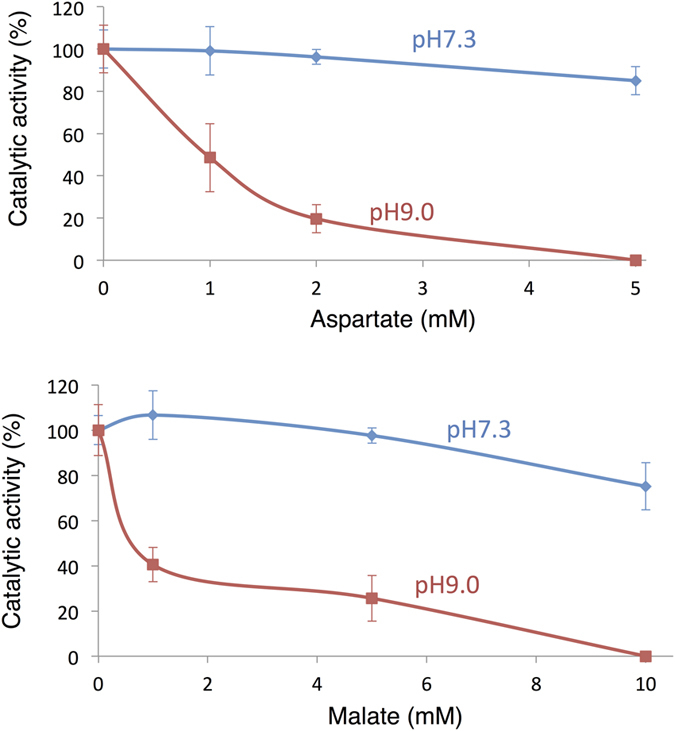
*Sy*PEPC activity at pH 7.3 and pH 9.0 in the presence of aspartate (top) or malate (bottom). The graphs show means ± SD obtained from three independent experiments. The activity of *Sy*PEPC in the absence of aspartate or malate was set at 100%.

We tested the inhibitory effects of aspartate and malate at alkaline pH, because the inhibitory effect on *Thermosynechococcus vulcanus* PEPC was stronger at alkaline pH than at neutral pH^15^. The inhibitory effects of malate and aspartate on *Sy*PEPC were enhanced at pH 9.0 compared with pH 7.3 (Fig. 3).

### *In silico* prediction and biochemical assay identified a glutamate residue at position 954 as important for allosteric regulation

**Figure 4:**
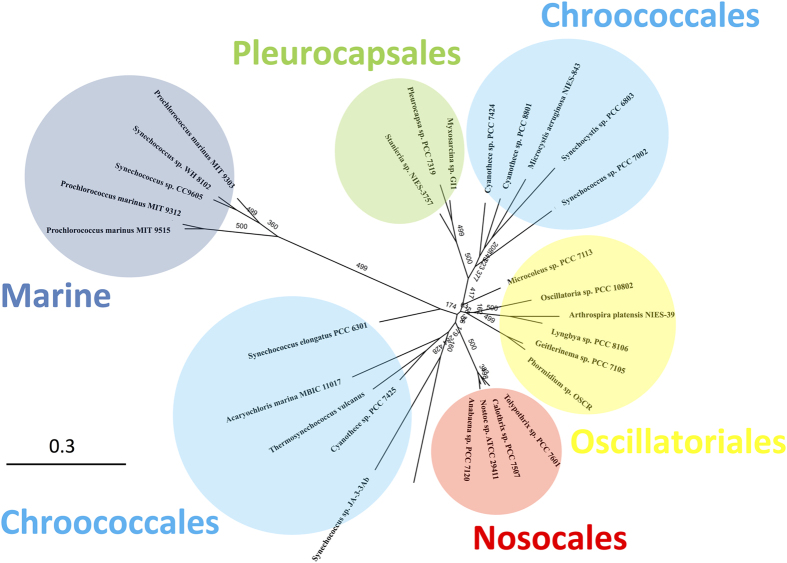
Phylogenetic analysis of the PEPCs from cyanobacteria, *Flaveria, Zea mays*, and *E. coli*. Protein sequences and accession numbers were obtained from GenBank. The protein sequences were aligned by the software CLC Sequence Viewer, and a maximum-likelihood tree based on 780 conserved amino acids was constructed using PHYML online (http://www.atgc-montpellier.fr/phyml/). The bootstrap values were obtained from 500 replications.

To understand the differences among cyanobacterial PEPCs, phylogenetic analysis was performed. The phylogenetic tree of PEPCs built using maximum likelihood methods showed a classification dependent on order; the PEPCs of *Synechocystis* 6803, *Thermosynechococcus vulcanus*, and *Coccochloris peniocystis*, all three of which belong to the order *Chroococcales*, were grouped in the same cluster, and were distinguished from *Anabaena* 7120 belonging to the order *Nostocales* (Fig. 4).

**Figure 5:**
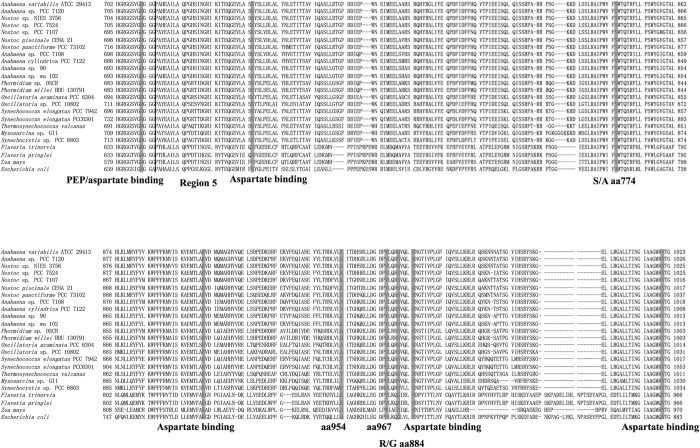
Multiple protein sequence alignment of phosphoenolpyruvate carboxylase. Only the alignment of region 5 (carboxyl terminal region involved in allosteric regulation of PEPCs) is shown in this figure. The multiple sequence alignment was performed using CLC Sequence Viewer.

A previous biochemical analysis showed that *Anabaena* 7120 PEPC (hereafter *An*PEPC) is sensitive to aspartate and malate^12^, but *Sy*PEPC was less sensitive to these metabolites (Table 1). To reveal the cause of the difference among these cyanobacterial PEPCs, a multiple sequence alignment was performed with the software CLC sequence viewer 7.0 (Fig. 5). The carboxyl-terminal region, called region 5, is important for inhibitor binding in higher plants^7^,^18^, and five conserved amino acid residues are important for aspartate inhibition^11^ (Fig. 5). These amino acid residues were also conserved in cyanobacterial PEPCs (Fig. 5). Therefore, at least one other amino acid residue is responsible for the difference between cyanobacterial and higher plant PEPCs. We first looked for amino acid residues unique to *Sy*PEPC and found 28 (Fig. 5). Among them, we searched for amino acid residues that were highly conserved in the order *Nostocales (Nostoc*/*Anabaena*) but different from those in either *Oscillatoriales* or *Chroococcales* (including *Synechococcus* and *Synechocystis*). Consequently, we found two candidates—the amino acids at positions 954 and 967 in *Sy*PEPC, which were glutamate and serine, respectively (Fig. 5).

**Figure 6:**
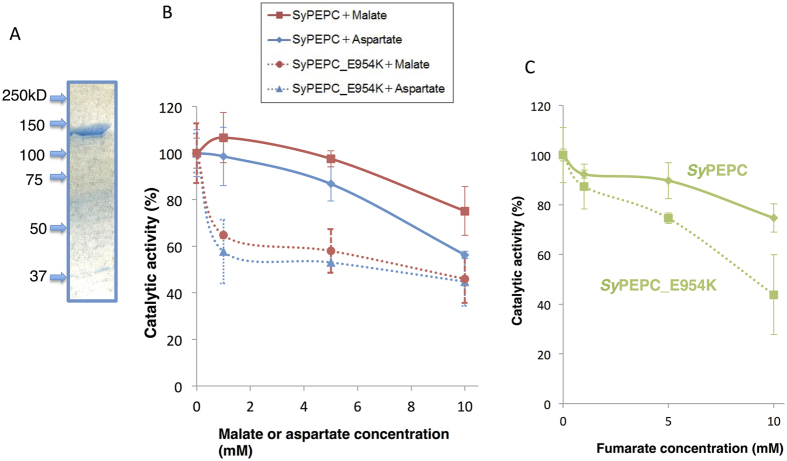
Biochemical analysis of *Sy*PEPC with a single substituted amino acid residue. *Sy*PEPC_E954K is *Sy*PEPC with the glutamate at position 954 substituted with lysine. (**A**) Purification of GST-tagged *Sy*PEPC_E954K. Proteins were electrophoresed on an 8% SDS-PAGE gel, and stained with Instant Blue reagent. Arrowheads indicate the molecular weight. (**B**) Effect of malate on *Sy*PEPC_E954K activity. Data represent means ± SD of relative activity from three independent experiments. *Sy*PEPC activity in the absence of malate was set at 100%. (**C**) Effect of fumarate on *Sy*PEPC_E954K activity. The data represent means ± SD of relative activity from three independent experiments. The *Sy*PEPC activity in the absence of fumarate was set at 100%.

Because the PEPCs in the order *Nostocales* contained lysine at position 954 and valine at position 967, we substituted the glutamate residue at position 954 in *Sy*PEPC with lysine (the protein was named *Sy*PEPC_E954K) and the serine residue at position 967 with valine (*Sy*PEPC_S967V). Biochemical analysis revealed that *Sy*PEPC_S967V had no enzymatic activity, but purified *Sy*PEPC_E954K (Fig. 6A) had enzymatic activity. *Sy*PEPC_E954K activity was reduced to 60% in the presence of 1 mM aspartate or malate (Fig. 6B), although neither 1 mM aspartate nor malate markedly decreased *Sy*PEPC activity (Fig. 6B). The addition of 5 mM aspartate or malate showed similar results to 1 mM on *Sy*PEPC and *Sy*PEPC_E954K (Fig. 6B). The *V*_max_ value of *Sy*PEPC_E954K was increased to 2.2 units/mg. The *K*_m_ value of *Sy*PEPC_E954K for PEP (0.82 mM) was more than double that of *Sy*PEPC, but the *K*_m_ value for HCO_3_^−^ (0.76 mM) was not altered. The inhibitory effect of fumarate was also enhanced in *Sy*PEPC_E954K compared with *Sy*PEPC (Fig. 6C).

### A conserved lysine residue in *Anabaena* 7120 PEPC is important for allosteric regulation

**Figure 7:**
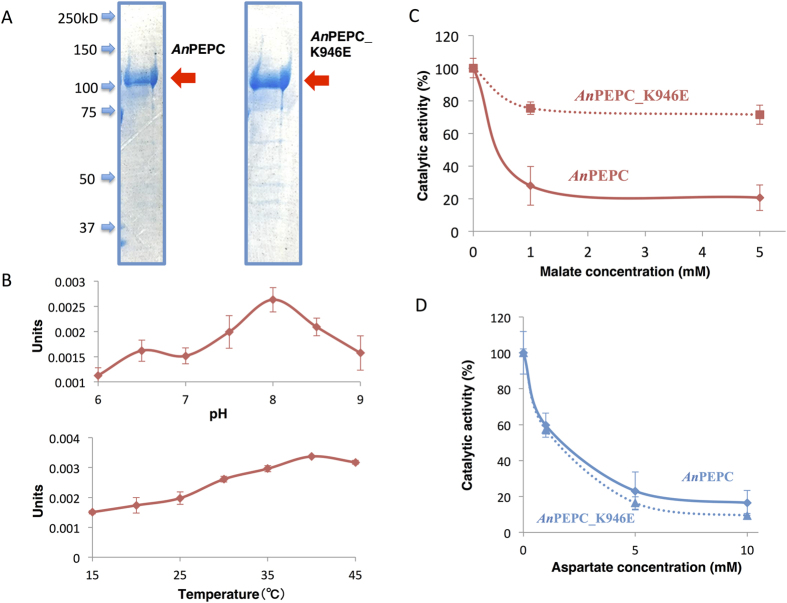
Biochemical analysis of *Anabaena* 7120 PEPCs (*An*PEPC). (**A**) Purification of GST-tagged *An*PEPC and *An*PEPC_K946E (the lysine residue was substituted with glutamate). Proteins were electrophoresed on an 8% SDS-PAGE gel, and stained with Instant Blue reagent. Arrowheads indicate the molecular weight. (**B**) Effect of temperature and pH on *An*PEPC activity. Data represent relative values of means ± SD from three independent experiments. Sixteen pmol (0.6 μg) of *Sy*PEPC was used for the enzyme assay. One unit of PEPC activity was defined as the consumption of 1 μmol NADPH per minute. (**C**) Effect of malate on *An*PEPC_K946E activity. Data represent means ± SD of relative activity from three independent experiments. *An*PEPC activity in the absence of malate was set at 100%. (**D**) Effect of aspartate on *An*PEPC_K946E activity. The data represent means ± SD of relative activity from three independent experiments. The *An*PEPC activity in the absence of aspartate was set at 100%.

The importance of the amino acid residue at position 954 in *Sy*PEPC was then examined in another cyanobacterium, *Anabaena* 7120. The lysine residue at position 946 in *An*PEPC is equivalent to the glutamate residue at position 954 in *Sy*PEPC. We substituted lysine 946 of *An*PEPC with glutamate, and named the protein *An*PEPC_K946E. Both GST-tagged *An*PEPC and *An*PEPC_K946E were similarly purified by affinity chromatography (Fig. 7A). The optimum pH and temperature of *An*PEPC were 8.0 and 35 °C (Fig. 7B). The activity of *An*PEPC in the absence or presence of either malate or aspartate was determined at various PEP concentrations (Fig. S2). A biochemical assay demonstrated that *An*PEPC_K946E was less inhibited by malate (the activity decreased to 80% in the presence of 1 mM malate) than *An*PEPC, the activity of which decreased to less than 30% in the same conditions (Fig. 7C). Additionally, 5 mM malate had a similar effect to 1 mM malate on both *An*PEPC and *An*PEPC_K946E (Fig. 7C). The inhibitory effect of aspartate on *An*PEPC was not altered by this amino acid substitution (Fig. 7D). The *V*_max_ values of *An*PEPC and *An*PEPC_K946E were 2.6 and 3.6 units/mg, respectively. The *K*_m_ values of *An*PEPC and *An*PEPC_K946E for PEP were 1.1 and 0.8 mM, respectively. The *K*_m_ values of *An*PEPC and *An*PEPC_K946E for HCO_3_^−^ were 0.24 and 0.25 mM, respectively.

## Discussion

In this study, we demonstrated the biochemical properties of *Sy*PEPC, which are unique among cyanobacterial PEPCs. Other groups showed that the optimum pH and temperature of the PEPCs in *Thermosynechococcus vulcanus* and *Coccochloris peniocystis* are pH 9.0 and 42 °C, and pH 8.0 and 40 °C, respectively^14^,^15^. The optimum pH of cyanobacterial PEPCs is thus 7.0–9.0; *Sy*PEPC is relatively active at acidic pH and low temperature (Fig. 1B and C). The optimum pH of C4-type PEPCs from *Sorghum, Digitaria sanguinalis*, and *Zea mays* is 7.0–8.0^15^,^19^,^20^, and therefore the optimum pH of *Sy*PEPC is similar to C4-type plants (Fig. 1B). *In silico* analysis provided the aliphatic index (Ai), which was calculated from the ratio of alanine, valine, isoleucine, and leucine in the primary amino acid sequence^21^. High Ai values suggest proteins are highly stable over a large range of temperatures. The Ai values of the PEPCs in *Nostocales* are higher than in *Chroococcales*^21^, and the *in silico* prediction is consistent with our results; *An*PEPC is more active at high temperature than *Sy*PEPC (Figs 1B and 7B). The combination of *in silico* and biochemical analyses thus drives the development of PEPC studies in cyanobacteria, as also shown in the multiple alignment and phylogenetic tree (Figs 4 and 5).

The *K*_m_ value of *Sy*PEPC for PEP was 0.34 mM (Fig. 2), which is close to the *K*_m_ value of PEPCs of *Thermosynechococcus vulcanus* (0.58 mM)^15^. The *K*_m_ value of *An*PEPC for PEP (1.1 mM) was higher than those of unicellular cyanobacteria, demonstrating the apparent distinction of PEPC kinetics between the orders *Chroococcales* and *Nostocales*. The *K*_m_ values for PEP of the PEPCs in *Oryza sativa* and *Flaveria pringlei* (C3-plants) are 0.03–0.56 mM and those of PEPCs in *Flaveria trinervia* and *Zea mays* (C4-plants) are 0.28–1.5 mM^22^,^23^,^24^. The *K*_m_ value for PEP of *Sy*PEPC is thus in between C3- and C4-plants. In the case of PEPCs of *Flaveria* species, the increased PEP saturation kinetics depends on a serine residue at position 774^22^. Our data revealed that the amino acid at positions 954 in *Sy*PEPC and 946 in *An*PEPC affect the *K*_m_ values for PEP, but not for bicarbonate. These results indicate the residue important for the binding of PEP to PEPC is different from that in higher plants. The *K*_m_ value for bicarbonate of *Sy*PEPC (0.8 mM) was higher than those of PEPCs in both C3- and C4-plants (between 0.06 and 0.33 mM)^23^. These results may indicate the necessity for a carbon concentration mechanism in cyanobacteria to support carbon fixation by encapsulation of Rubis CO_2_. Phylogenetic analyses revealed that the kinetic changes of *Flaveria* PEPCs occurred during the last steps of the evolutionary process^7^, and the variation among cyanobacterial PEPCs may also have appeared during recent evolution.

We found that *Sy*PEPC was less inhibited by metabolic effectors, and that a single amino acid substitution at position 954 affected the allosteric regulation by malate or aspartate (Fig. 6B). The inhibitory effect of the metabolites on *Sy*PEPC was higher at pH 9.0 than at pH 7.3 (Fig. 3), while the optimal enzymatic activity was at pH 7.3 (Fig. 1C). In *Coccochloris peniocystis*, PEPC activity is higher at pH 8.0 than at pH 7.0, while the inhibitory effect of aspartate or malate is greater at pH 7.0 than at pH 8.0^14^. Thus, the optimal pHs for enzymatic activities and inhibitory effects by metabolites are not correlated in cyanobacteria. The importance of the amino acid substitution between glutamate and lysine was conserved in another cyanobacterium, *Anabaena* 7120 (Fig. 7C). Among *Flaveria* species, *F. pringlei* performs C3-type photosynthesis and *F. trinervia* performs C4-type photosynthesis^9^,^25^,^26^. The C3-type PEPCs in *Flaveria* containing an arginine residue at position 884 are inhibited by malate, while the C4-type PEPCs containing a glycine residue at position 884 are tolerant to malate^18^. Our multiple sequence alignment analysis revealed the amino acid residue at position 954 in *Sy*PEPC is not equivalent to the residue at position 884 in *Flaveria* PEPCs (Fig. 5). The lysine residue at position 946 in *Anabaena* is highly conserved among nitrogen-fixing cyanobacteria (Fig. 5), and the positive charge of lysine may play critical role in malate binding. The inhibitory effect of aspartate was not affected by substitution of the lysine residue at position 946 in *An*PEPC (Fig. 7D). At least five amino acid residues play roles in the binding of aspartate to PEPC proteins^15^ (Fig. 5); therefore, other amino acids compensate for the absence of the lysine residue at position 946 in *An*PEPC during aspartate binding. Thus, we discovered changes in allosteric regulation by a single amino acid substitution are conserved in both cyanobacteria and higher plants, although the key residues differ. In this study, we focused on region 5 of cyanobacterial PEPCs and showed the importance of this region in allosteric regulation. The structure of cyanobacterial PEPCs remains to be determined and future biochemical studies will elucidate the detailed mechanism of allosteric inhibition in cyanobacterial PEPCs.

## Methods

### Construction of cloning vectors for recombinant protein expression

The region of the *Synechocystis* 6803 genome containing the *ppc* (sll0920, encoding *Sy*PEPC) ORF was amplified by PCR using KOD plus neo polymerase and the primers 5′-GAAGGTCGTGGGATCATGAACTTGGCAGTTCCTG-3′ and 5′-GATGCGGCCGCTCGAGTCAACCAGTATTACGCATTC-3′. The amplified DNA fragments were cloned into the *Bam*HI-*Xho*I site of pGEX5X-1 (GE Healthcare Japan, Tokyo, Japan) using an In-Fusion HD cloning kit (Takara Bio, Shiga, Japan). Site-directed mutagenesis was commercially performed by Takara Bio. For *Sy*PEPC_E954K and *Sy*PEPC_S967V, +2860–2862 and +2899–2901 from the start codon were changed from GAA to AAA and from TCT to GTG, respectively.

The region of the *Anabaena* 7120 genome containing the *ppc* (all4861, encoding *An*PEPC) ORF was artificially synthesized and cloned into the *Bam*HI-*Xho*I site of pGEX5X-1 by Takara Bio.

### Affinity purification of recombinant proteins

The expression vectors were transformed into *E. coli* BL21(DE3) (Takara Bio). Several liters of *E. coli* containing the vectors were cultivated at 30 °C by shaking (150 rpm), and protein expression was induced overnight by adding 0.01 mM isopropyl β-D-1-thiogalactopyranoside (Wako Chemicals, Osaka, Japan).

Affinity chromatography for protein purification was performed as described previously^27^. Briefly, *E. coli* cells from 2 L culture were disrupted by sonication VC-750 (EYELA, Tokyo, Japan) for 5 min with 30% intensity, and centrifuged at 5,800 × *g* for 2 min at 4 °C. The supernatant was transferred to a new 50-mL plastic tube, and 560 μL of glutathione-Sepharose 4B resin (GE Healthcare Japan, Tokyo, Japan) was added. After rotating for 30 min, the resin was washed with 500 μL of PBS-T (1.37 M NaCl, 27 mM KCl, 81 mM Na_2_HPO_4_·12H_2_O, 14.7 mM KH_2_PO_4_, 0.05% Tween-20) with 1 mM ATP, and eluted three times with 500 μL of GST elution buffer (50 mM Tris-HCl, pH 8.0, 10 mM reduced glutathione). The protein concentration was measured with a PIERCE BCA Protein Assay Kit (Thermo Scientific, Rockford, IL). Protein purification was confirmed by SDS-PAGE with staining with InstantBlue (Expedion Protein Solutions, San Diego, CA).

### Enzyme assay

For the assay of the purified proteins, 4 pmol of *Sy*PEPCs or 16 pmol of *An*PEPCs were mixed in a 1 mL assay solution (100 mM MOPS-Tris, 10 mM MgCl_2_, 1 mM EDTA, 50 mM NaHCO_3_, 0.2 mM nicotinamide adenine dinucleotide hydride (NADH), 2.5 mM PEP, 10 U of malate dehydrogenase (Oriental Yeast, Tokyo, Japan)). For the cell extract assay, 150 μg of total proteins was added to 1 mL assay solution. The absorbance at *A*_340_ was measured using a Hitachi U-3310 spectrophotometer (Hitachi High-Tech., Tokyo, Japan). One unit of PEPC activity was defined as the consumption of 1 μmol NADPH per minute. *V*_*max*_ and *K*_*m*_ values were determined by a Lineweaver-Burk double reciprocal plot. The results were plotted as a graph of the rate of reaction against the concentration of substrate. The Y and X intercepts were 1/*V*_*max*_ and −1/*K*_*m*_, respectively.

### Bacterial strains

The glucose-tolerant (GT) strain of *Synechocystis* sp. PCC 6803, isolated by Williams^28^, and *Nostoc* sp. PCC 3756 from the National Institute of Environmental Science (Tsukuba, Japan) were grown in modified BG-11 medium, consisting of BG-11_0_ liquid medium^20^ supplemented with 5 mM NH_4_Cl (buffered with 20 mM HEPES–KOH, pH 7.8). The liquid cultures were bubbled with air containing 1% (v/v) CO_2_ (flow rate was 20–50 mL/min) and incubated at 30 °C under continuous white light (~50–70 μmol photons m^−2^ s^−1^). For enzymatic assay, the cells were suspended in 1 mL of assay solution with one-tenth of a tablet of Complete mini protease inhibitor (Roche, Basel, Switzerland), followed by disruption with a VC-750 sonicator (EYELA) for 3 min with 30% intensity. The cell extracts were centrifuged at 5,800 × *g* for 2 min at 4 °C, and the supernatant was used for PEPC activity assay.

### Statistical analysis

*P*-values were determined using paired two-tailed Student’s *t*-tests with Microsoft Excel for Mac 2011 (Redmond, WA, USA). All results were obtained using biologically independent replicates.

## Additional Information

**How to cite this article**: Takeya, M. *et al*. Allosteric Inhibition of Phosphoenolpyruvate Carboxylases is Determined by a Single Amino Acid Residue in Cyanobacteria. *Sci. Rep.* **7**, 41080; doi: 10.1038/srep41080 (2017).

**Publisher's note:** Springer Nature remains neutral with regard to jurisdictional claims in published maps and institutional affiliations.

## Supplementary Material

Supplemental Information

## Figures and Tables

**Table 1:** Effect of various metabolites on *Sy*PEPC activity.

Compounds	*Sy*PEPC activity (*in vitro*)
Mock	100 ± 5.2
GTP	101 ± 0.6
Acetyl-CoA	111 ± 14.0
Fructose-1,6-bisphophate	96.9 ± 4.1
Aspartate	85.2 ± 10.7
Citrate	86.1 ± 6.7
Malate	77.1 ± 6.3
Fumarate	75.0 ± 9.7
Succinate	124 ± 12.3

Enzyme activities were measured at pH 7.3 and 30 °C in the presence of 0.5 mM PEP. The concentration of each metabolite was 1 mM, except for GTP (5 mM), acetyl-CoA (0.4 mM), and fructose-1,6-bisphosphate (2 mM). Mock indicates the enzymatic activity in the absence of additional compounds. Data represent means ± SD from three independent assays. Mock was set at 100%.
