# Supplementary material for: Allosteric Inhibition of Phosphoenolpyruvate Carboxylases is Determined by a Single Amino Acid Residue in Cyanobacteria
Source: Sci Rep. 2017 Jan 24;7:41080. doi: 10.1038/srep41080 (PMC5259782; doi:10.1038/srep41080)
Supplement: Supplemental Information [file srep41080-s1.pdf]

Allosteric Inhibition of Phosphoenolpyruvate Carboxylases Is Determined by a Single Amino Acid Residue in Cyanobacteria

Masahiro Takeya[1], Masami Yokota Hirai[2], Takashi Osanai[1,2*]

[1]School of Agriculture, Meiji University, 1-1-1, Higashimita, Tama-ku, Kawasaki, Kanagawa 214-8571, Japan

[2]RIKEN Center for Sustainable Resource Science, 1-7-22 Suehiro-cho, Tsurumi-ku, Yokohama, Kanagawa 230-0045, Japan

Address correspondence to: Dr. Takashi Osanai, Meiji University. 1-1-1 Higashimita, Tama-ku, Kawasaki, Kanagawa 214-8571, Japan

Tel: +81-44-934-7103. Fax: +81-44-934-7103. E-mail: tosanai@meiji.ac.jp

Running Title: Allosteric Inhibition of PEPC in Cyanobacteria

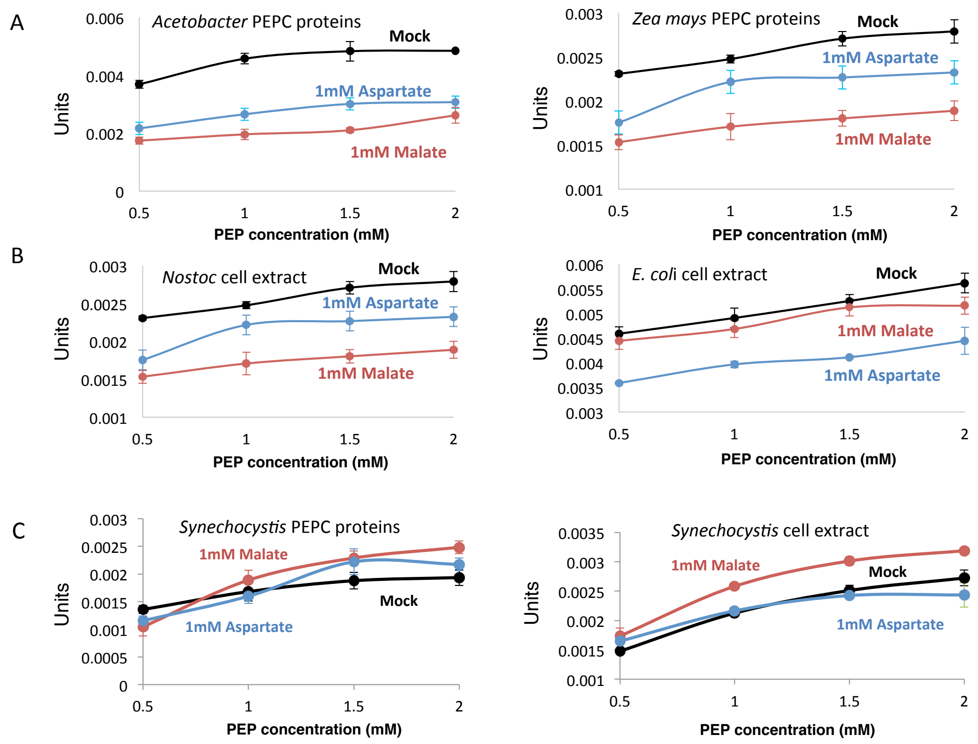

Figure S1. (A) The activities of commercially available PEPC proteins. The left and right graphs show the activities of purified PEPCs from *Acetobacter* (5 μg) and *Zea mays* (2.5 μg), respectively. The activity was measured in the absence or presence of 1 mM malate and aspartate at various PEP concentrations. The graphs show means ± SD obtained from three independent experiments. (B) The activities of PEPCs in cell extracts from *Nostoc* sp. NIES-3756 (total 1 mg proteins) (left) and *E. coli* DH5α (total 100 μg proteins) (right). The graphs show means ± SD obtained from three independent experiments. Mock indicates the enzymatic activity in the absence of additional compounds. (C) The activities of *Synechocystis* 6803 PEPC from purified proteins (0.6 μg) (left) and cell extracts (total 250 μg proteins) (right). The data for the purified proteins were derived from Fig. 2A. One unit of PEPC activity was defined as the

consumption of 1 μmol NADPH per minute.

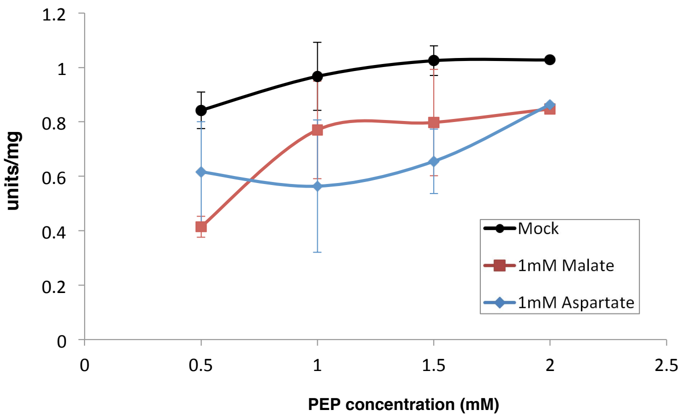

Figure S2. Saturation curves of the activity of purified *An*PEPC. The graph shows the means of three independent experiments. Mock indicates the enzymatic activity in the absence of additional compounds. One unit of PEPC activity was defined as the consumption of 1 μmol NADPH per minute.
